# Allele Frequency of ABO Blood Group Antigen and the Risk of Esophageal Cancer

**DOI:** 10.1155/2014/286810

**Published:** 2014-06-19

**Authors:** Narender Kumar, Akhil Kapoor, Ashok Kalwar, Satya Narayan, Mukesh Kumar Singhal, Akhender Kumar, Abhishek Mewara, Megh Raj Bardia

**Affiliations:** ^1^Sardar Patel Medical College, Bikaner 334003, India; ^2^Department of Radiation Oncology, Sardar Patel Medical College, Bikaner 334003, India; ^3^Medical Oncology Division, Department of Oncology, Sardar Patel Medical College, Bikaner 334003, India; ^4^Medical College & S.S.G. Hospital, Vadodara 390001, India; ^5^Government Medical College, Kota 324005, India

## Abstract

*Background*. ABO blood group and risk of squamous cell carcinoma of esophagus have been reported by many studies, but there is no discipline that had provided association with the genotype and gene frequency by population statics. *Methods*. We conducted a case-control study on 480 patients with squamous cell carcinoma of the esophagus and 480 noncancer patients. ABO blood group was determined by presence of antigen with the help of monoclonal antibody. Chi-square test and odds ratio (OR) with 95% confidence intervals (CIs) were calculated by statistical methods, and gene frequencies were calculated by Hardy-Weinberg model. *Results*. We observed significant associations between ABO genotype and squamous cell carcinoma of esophagus. OR (95% CIs) was 1.69 (1.31–2.19) for presence of B antigen allele relative to its absence (*P* < 0.0001); in female subgroup OR (95% CIs) observed at 1.84 (1.27–2.65) was statistically significant (*P* = 0.001). SCC of esophagus shows significant difference in comparison to general population; blood group B is found to be higher in incidence (*P* = 0.0001). Increased risk of cancer was observed with absence of Rh antigen (*P* = 0.0001). Relatively increased gene frequency of *q*[B] allele is observed more significantly in female cancer patients (*P* = 0.003). *Conclusion*. Statistically significant association between squamous cell carcinoma of the esophagus and ABO and Rh genotype is identified by this study. Sex and anatomical site of cancer also present with statistically significant relative association. However, larger randomised trials are required to establish the hypothesis.

## 1. Introduction

Phenotypic presentation of the organism is determined by its genotype structure. Blood group since the discovery has been linked with many diseases though the explanation for the association between ABO blood groups and some diseases is still not understood. The genetic composition of the ABO blood type is an easily accessible component. Numerous other reports have documented a relative association between susceptibility to cancer and blood group type. Blood group A presents with high incidence in various cancers of salivary gland, colon, uterus, ovary, kidney, and neurologic tumors [1], and O blood group has an association with skin and melanoma [2]. B blood group is associated with oesophageal cancer [3]. The increased number of pancreatic cancers among the patients with non-O blood type with an increased incidence of B blood type as compared to control has been reported [4].

The ABO blood group system was the first genetic polymorphism discovered in humans. It consists of three alleles: two codominant A and B alleles and one silent and recessive O allele. The system is controlled by a single gene at the ABO locus at 9q34 region of the chromosome. This gene encodes a glycosyltransferase enzyme that adds a sugar residue to a carbohydrate structure known as the H antigen that is present in the membrane of red cells as well as most epithelial and endothelial cells. The A allele codes for an enzyme that adds an N-acetyl galactosamine to the H antigen, while the B allele, which differs from the former by four amino acid changes, codes for an enzyme that adds a D-galactose. The O allele occurs most frequently in modern humans and carries a human-specific inactivating mutation which produces a nonfunctional enzyme, and thus the H antigen remains without further modification on the surface of the cells [5, 6]. The Rhesus factor is clinically the most important protein-based blood group system. With 49 antigens so far described, it is the largest of all 29 blood group systems. The antigens are located on two Rhesus proteins—RhD and RhCE—and are produced by differences in their protein sequences. In CD nomenclature, they are termed CD240D and CD240CE. Unlike proteins of other blood groups, Rhesus proteins are expressed only in the membranes of red blood cells and their immediate precursors [7].

Genetic alteration of this region is common in many cancers. Expression of antigen depends on the activity of the particular gene. Thus, the blood group antigen expression may be affected by the nature of genetic change in cell. Blood group gene expression that presents with a relative correlation in various tumors with metastasis and prognosis has been reported for various human malignancies, such as prostate, colon, breast, and prostate cancer. As the blood group is determined by the presence of antigens and these antigens are glycoproteins, which are expressed on the cell surface and function as cell adhesion molecules. The loss or presence of blood group antigens can increase cellular motility or facilitate the interaction between tumor cells and endothelial cells of distant organs [8–11]. The deficiency of A or B epitope in many cancers has been reported, which is associated with accumulation of their precursor, which causes enhanced malignancy, though the molecular genetics mechanism leading to such phenotypic changes is unclear. The expression of certain blood group carbohydrate antigens on the surface of cancer cells thus can be regarded as an end product of tumor progression that can be used as useful prognostic and diagnostic markers [12–14].

The ABO blood group frequency distribution varies in different geographical and ethnic groups and socioeconomic groups [15]. In India, the ABO blood group frequency is variable; the frequency for B ranges from 6% in Negritos of Andamans to 48% in Birijas of Bihar, while A group is 20–30% in Western and Eastern Himalayas [16]. The state of Punjab in North-West India is inhabited by a mixed population of Caucasian and Indoscythian racial stock, and the blood group frequency in North-West Indian population is B (35–39%) > O (30.0–34.0%) > A (20–24%) > AB (8.0–10.0%) [17, 18]. The current study was undertaken to correlate ABO blood group frequency with squamous cell carcinoma of esophagus in this region to evaluate the utility of ABO blood group as a preclinical marker.

## 2. Materials and Methods

A case control study design was used with collection of data for age, sex, ABO, and Rh blood type with the inclusion criteria for case being the squamous cell carcinoma of the esophagus in the histology at Acharya Tulsi Regional Cancer Treatment and Research Centre, Sardar Patel Medical College, Bikaner (India), from January 2013 to December 2013. The control sample was collected from donors at the blood bank of the Department of Transfusion Medicine. A total of 480 cancer patients (252 males and 228 females) and 480 healthy controls (240 males and 240 females) were obtained for the correlation with ABO blood groups and Rh antigen status. Slide agglutination test and blood group of the patient and control were accessed. Anti-A, Anti-B, and Anti-D (IgM) monoclonal antibodies were used for screening of blood group (Mediclone Biotech, Chennai, India). Data is stratified by sex, histology, and anatomical site of cancer of esophagus. The blood group frequencies were compared by Chi-square test and odds ratio (OR) with 95% confidence intervals (CIs) being calculated using SPSS software for windows version 20.0 (Armonk, NY, IBM Corp.). The gene and allele frequencies of blood group were calculated by Hardy-Weinberg model (Table 5) using S2 ABO estimator software (Silvasquare, Lisboa, Portugal).

## 3. Results

The distribution of blood groups in the esophageal squamous cell carcinoma patients (*n* = 480) was 98 (20.4%) blood group A, 58 (12.1%) blood group AB, 226 (47.1%) blood group B, and 98 (20.4%) blood group O. Rh status was positive in 424 (88.3%) and negative in 56 (11.7%) patients (Table 1). Overall, blood group B is the most prevalent blood group in general population and patients with squamous cell carcinoma of esophagus. There were statistically significant differences in the distribution of ABO blood groups among patients and general population (*χ*2 = 20.296, *P* = 0.0001). In the patient group, the frequencies of blood groups B and AB were more and for blood groups A and O they were less than the control group. These findings were also seen in female subgroup (*χ*2 = 17.177, *P* = 0.0006). However in male subgroup, the distribution of ABO blood groups did not significantly differ between cases and controls (*χ*2 = 9.611, *P* = 0.0221). Analyzing the blood group distribution on the basis of anatomical site of the cancer by dividing the esophagus into the upper, middle, and lower parts, squamous cell carcinoma of lower part of the esophagus shows significant difference in comparison to the general population, and blood group B is found to be higher in incidence (*χ*2 = 12.06, *P* = 0.007) (Table 2).

There were also statistically significant differences in the status of Rh blood groups among patients and general population (*χ*2 = 13.964, *P* = 0.0001). Presence of Rh antigen was about 6.7% less prevalent in squamous cell carcinoma of esophagus patients (88.3%) in comparison to general population (95%). The female subgroup shows 2.9% less prevalence of Rh antigen in comparison to the male subgroup. Rh antigen status does not show any statistically significant difference in distribution according to cancer site in comparison to general population.

In comparison, of individuals with the presence of B antigen (blood groups B and AB) and without B antigen (blood groups A and O), this difference became more prominent (*P* < 0.0001) (odds ratio = 1.69, 95% CI: 1.31–2.19). These findings were also seen in female and male subgroups; female subgroup shows statistically more significance. Female subgroup presents with (*P* = 0.001) (odds ratio = 1.84, 95% CI: 1.27–2.65) statistically more significant than male subgroup (*P* = 0.012) (odds ratio = 1.57, 95% CI: 1.10–2.24) (Table 3).

The gene frequencies [*q*] of blood group antigen B were 0.362 in cancer patients, which was higher than that of the control population (0.265), and the relative risk B : O was 1.69 with statistical significance. On subgroup analysis, female patients of oesophageal squamous cell carcinoma present with a gene frequency [*q*] of 0.376 for the B antigen of blood group, which is statistically significant (*P* = 0.003). Patients with carcinoma of the lower third esophagus present with a higher gene frequency [*q*] (0.372) for blood group antigen B in comparison to upper and middle third of the esophagus (Table 4). This suggests that individuals with blood group antigen B are more susceptible to squamous cell carcinoma of esophagus.

Homozygous or heterozygous trait is determined by the presence of allele type. Genotype of blood group A (AA + AO), B (BB + BO), AB (AB), and O (OO) alleles. There were not much significant results according to the homo- or heterozygous stratification.

## 4. Discussion

In cancer patients, the relative incidence of B blood group is more frequent in squamous cell carcinoma of esophagus. Cancer patients with presence of B antigen (B and AB blood group) were higher, whereas in controls, absence of B antigen (A and O blood groups) was in higher frequency. In previous studies, contradictory reports are available about the association of esophageal cancer with any blood group. Increased B blood group in the esophageal squamous cell carcinoma and increased O blood group for adenocarcinoma [19] of esophagus have been reported. In esophageal cancer patients, 33.1% blood group A, 31.7% blood group O, 25.9% blood group B, and 9.3% blood group AB were reported. Rh antigen was present in 92% and absent in 8% of the patients [20]. The relative increase in the frequency of blood type B as compared to control has been reported in esophageal cancer. These results suggest that the presence of B antigen plays a role in the development of the esophageal cancer by the susceptible genetic mutation in the vicinity of the locus of blood group genes that involves various etiological mechanisms. The hypothesis for this association of squamous cell carcinoma of esophagus with the presence of B antigen (B + AB blood group) is based on the grounds that it can protect the tumor cells by masking the immune system for the cancer cell that presents with an antigen similar to that of B antigen of blood group.

Hardy-Weinberg principle states that allele and genotype frequencies in a population will remain constant from generation to generation in the absence of other evolutionary influences. The frequency of* q*[B] allele of blood group in the squamous cell carcinoma patients is increased and that of* r*[O] allele is decreased relative to the control population; this suggests that genetic changes at the locus for B antigen allele have risks while the absence of both A and B antigen alleles is associated with reduced risk for cancer development.

The homotypic and heterotypic cell adhesion mediated by interactions of certain blood group carbohydrates with corresponding lectins are a critically important event at the extravasation step of the metastatic cascade when metastatic cancer cells escape from circulation into distant sites of secondary tumor growth. People with blood groups B and AB lack antibodies to B and so are more prone to develop these carcinomas [1]. Deletion or reduction of histoblood group A or histoblood group B antigen in tumors of A or B individual is correlated with the degree of malignancy and metastatic potential in many types of human cancers.

The cancers of different anatomical sites and histology show variable positive or negative correlation with the blood group. Distribution of blood groups in the racial and ethnic groups and the sample size play an important role in determining the goodness of the interpretation of the risk of cancer development. To estimate the individual patient's risk, the blood type and genetic composition of the patient may be considered together along with other risk factors. The recognition of genetic and environmental factors amongst racial and ethnic groups may offer insights into the observed epidemiological patterns and thus provide better understanding of the development and control of cancer.

## Figures and Tables

**Table 1:** Frequency of ABO blood group and Rh antigen.

	*n*	A	AB	B	O	Rh^+^	Rh^−^
All							
Cases	480	98 (20.4)	58 (12.1)	226 (47.1)	98 (20.4)	424 (88.3)	56 (11.7)
Control	480	106 (22.1)	43 (8.9)	178 (37.1)	153 (31.9)	456 (95)	24 (5)
Male							
Cases	252	48 (19)	41 (16.3)	106 (42.1)	57 (22.6)	228 (90.5)	24 (9.5)
Control	240	50 (20.9)	22 (9.1)	91 (37.9)	77 (32.1)	230 (95.8)	10 (4.2)
Female							
Cases	228	50 (21.9)	17 (7.5)	120 (52.6)	41 (18)	196 (86)	32 (14)
Control	240	55 (22.9)	22 (9.2)	86 (35.8)	77 (32.1)	226 (94.2)	14 (5.8)
Site of cancer (case)							
Upper	86	19 (22.1)	7 (8.1)	38 (44.2)	22 (25.6)	79 (91.9)	7 (8.1)
Middle	192	32 (16.7)	19 (9.9)	90 (46.9)	51 (26.5)	170 (88.5)	22 (11.5)
Lower	202	47 (23.3)	32 (15.8)	98 (48.5)	25 (12.4)	175 (86.6)	27 (13.4)

Values in parentheses are percentages.

**Table 2:** Chi-square test value and significance of ABO blood group and Rh status.

Cases ∗ control (*n*)	ABO blood group		Rh status	
	*χ* ^2^ test	*P* value	*χ* ^2^ test	*P* value
All	20.296	0.0001	13.964	0.0001
Male	9.611	0.0221	5.484	0.0191
Female	17.177	0.0006	8.874	0.0028
Site of cancer Case ∗ control (%)				
Upper	1.348	0.717	0.785	0.375
Middle	2.3447	0.484	2.791	0.094
Lower	12.06	0.007	4.223	0.039

Degree of freedom = 1.

**Table 3:** Presence of B antigen (B and AB blood groups) and absence of B antigen (O and A blood groups); Chi-square test with significance and odd ratio.

	*n*	Presence of B (B + AB)	Absence of B (O + A)	Chi-square	*P* value	Odds ratio	CI = 95%
All							
Cases	480	284 (59.17)	196 (40.83)	16.58	<0.0001	1.69	1.31–2.19
Control	480	221 (46.04)	259 (53.96)				
Male							
Cases	252	147 (58.33)	105 (41.67)	6.24	0.012	1.57	1.10–2.24
Control	240	113 (47.1)	127 (52.9)				
Female							
Cases	228	137 (60.1)	91 (39.9)	10.67	0.001	1.84	1.27–2.65
Control	240	108 (45)	132 (55)				
Site of cancer (case)							
Upper	86	45 (52.3)	41 (47.7)	0.212	0.645		
Middle	192	109 (56.77)	83 (43.23)	1.833	0.175		
Lower	202	130 (64.36)	72 (35.64)	8.248	0.004		

Degree of freedom = 1.

Values in parentheses are percentages.

**Table 4:** Gene frequency of allele with its significance.

Group	Gene frequency			Hardy-Weinberg log likelihood	*χ* ^2^	*P* value
	*p*[A]	*q*[B]	*r*[O]			
Control	0.169 (0.012)	0.265 (0.015)	0.565 (0.017)	−615.3477	0.002	0.964
Total EC	0.179 (0.013)	0.362 (0.017)	0.458 (0.018)	−604.5129	0.5856	0.444
Male	0.193 (0.018)	0.349 (0.024)	0.456 (0.025)	−331.8725	2.6853	0.101
Female	0.163 (0.018)	0.377 (0.026)	0.459 (0.027)	−271.8815	8.4190	0.003
Upper	0.166 (0.029)	0.312 (0.039)	0.521 (0.042)	−107.6611	0.7385	0.390
Middle	0.143 (0.018)	0.342 (0.027)	0.514 (0.029)	−237.0872	0.0039	0.950
Lower	0.222 (0.022)	0.408 (0.028)	0.369 (0.029)	−251.2710	1.2961	0.255

Values in parentheses are standard deviations.

**Table 5:** Hardy-Weinberg model for ABO blood group.

Phenotype (blood group)	Genotype	Phenotypic frequency	Genotypic frequency	Expected frequency
A	AA + AO	*n*A	*n*AA + *n*AO	*p* ^2^ + 2*pr *
B	BB + BO	*n*B	*n*BB + *n*BO	*q* ^2^ + 2*qr *
AB	AB	*n*AB	*n*AB	2*pq *
O	OO	*n*O	*n*OO	*r* ^2^

Hardy-Weinberg equation for ABO blood group is as follows:

*p* + *q* + *r* = 1,

*p* ^2^ + *q* ^2^ + *r* ^2^ + 2*pq* + 2*pr* + 2*qr* = 1.

## References

[1] Henderson J, Seagroatt V, Goldacre M (1993). Ovarian cancer and ABO blood groups. *Journal of Epidemiology and Community Health*.

[2] Karakousis CP, Evlogimenos E, Sun O (1986). Blood groups and malignant melanoma. *Journal of Surgical Oncology*.

[3] Aminian A, Mirsharifi R, Alibakhshi A, Khorgami Z, Dashti H, Hasani SM (2010). Relationship between esophageal cancer and blood groups. *World Applied Sciences Journal*.

[4] Nakao M, Matsuo K, Hosono S (2011). ABO blood group alleles and the risk of pancreatic cancer in a Japanese population. *Cancer Science*.

[5] Yamamoto F, Clausen H, White T, Marken J, Hakomori S (1990). Molecular genetic basis of the histo-blood group ABO system. *Nature*.

[6] Kermarrec N, Roubinet F, Apoil PA, Blancher A (1999). Comparison of allele O sequences of the human and non-human primate ABO system. *Immunogenetics*.

[7] Flegel WA, Wagner FF, Mueller-Eckhardt C, Kiefel V (2003). Blutgruppen: alloantigene auf erythrozyten. *Transfusionsmedizin*.

[8] Pack SD, Karkera JD, Zhuang Z (1999). Molecular cytogenetic fingerprinting of esophageal squamous cell carcinoma by comparative genomic hybridization reveals a consistent pattern of chromosomal alterations. *Genes Chromosomes Cancer*.

[9] Hu N, Roth MJ, Polymeropolous M (2000). Identification of novel regions of allelic loss from a genome wide scan of esophageal squamous cell carcinoma in a high risk Chinese population. *Genes Chromosomes Cancer*.

[10] Simoneau M, LaRue H, Aboulkassim TO, Meyer F, Moore L, Fradet Y (2000). Chromosome 9 deletions and recurrence of superficial bladder cancer: identification of four regions of prognostic interest. *Oncogene*.

[11] Zitzelsberger H, Engert D, Walch A (2001). Chromosomal changes during development and progression of prostate adenocarcinomas. *British Journal of Cancer*.

[12] Ichikawa D, Handa K, Hakomori S (1998). Histo-blood group A/B antigen deletion/reduction *vs.* continuous expression in human tumor cells as correlated with their malignancy. *International Journal of Cancer*.

[13] Sleeman JP, Kim U, LePendu J (1999). Inhibition of MT-450 rat mammary tumour growth by antibodies recognising subtypes of blood group antigen B. *Oncogene*.

[14] Le Pendu J, Marionneau S, Cailleau-Thomas A, Rocher J, Le Moullac-Vaidye B, Clement M (2001). ABH and lewis histo-blood group antigens in cancer. *Acta Pathologica, Microbiologica, et Immunologica Scandinavica*.

[15] Beardmore JA, Karimi-Booshehri F (1983). ABO genes are differentially distributed in socio-economic groups in England. *Nature*.

[16] Barua S (2002). *Human Genetics: An Anthropological Perspective*.

[17] Chandra T, Gupta A (2012). Frequency of ABO and rhesus blood groups in blood donors. *Asian Journal of Transfusion Science*.

[18] Verma P, Sachdeva SK, Verma KG, Saharan S, Sachdeva K (2013). Correlation of lip prints with gender, ABO blood groups and intercommissural distance. *North American Journal of Medical Sciences*.

[19] Caygill CP, Royston C, Charlett A (2011). Barrett’s, blood groups and progression to oesophageal cancer: is nitric oxide the link?. *European Journal of Gastroenterology and Hepatology*.

[20] Su M, Lu S-M, Tian D-P (2001). Relationship between ABO blood groups and carcinoma of esophagus and cardia in Chaoshan inhabitants of China. *World Journal of Gastroenterology*.

